# Microstructure Formation of Cement Mortars Modified by Superabsorbent Polymers

**DOI:** 10.3390/polym13203584

**Published:** 2021-10-18

**Authors:** Jan Fořt, Jiří Šál, Martin Böhm, María Jesús Morales-Conde, Manuel Alejandro Pedreño-Rojas, Robert Černý

**Affiliations:** 1 Department of Materials Engineering and Chemistry, Faculty of Civil Engineering, Czech Technical University in Prague, Thákurova 7, 166 29 Prague, Czech Republic; sal@mail.vstecb.cz (J.Š.); martin.bohm@fsv.cvut.cz (M.B.); cernyr@fsv.cvut.cz (R.Č.); 2 Institute of Technology and Business in České Budějovice, Okružní 517/10, 370 01 České Budějovice, Czech Republic; 3 Departamento de Construcciones Arquitectónicas 1, Escuela Técnica Superior de Arquitectura, Universidad de Sevilla, Avenida Reina Mercedes, n_ 2, 41012 Sevilla, Spain; mmorales@us.es; 4 Departamento de Urbanística y Ordenación del Territorio, Escuela Técnica Superior de Arquitectura, Universidad de Sevilla, Avenida Reina Mercedes, n_ 2, 41012 Sevilla, Spain; mpedreno@us.es

**Keywords:** cement mortar, superabsorbent polymer, porosity, mechanical strength, scanning electron microscopy, shrinkage

## Abstract

The utilization of superabsorbent polymers (SAPs) in cement-based materials has been found to be a promising means of mitigating the autogenous propagation of shrinkage and cracks. On the other hand, the undesired effects of SAPs’ application on functional properties, including mechanical strength, microstructure formation, and the evolution of hydration heat are not properly understood, given the variety in SAPs’ characteristics. To contribute to the present state-of-the-art, cement mortars, modified with two grades of SAPs by dosages of 0.3%, 0.6%, and 0.9%, were designed and studied with emphasis on the relationship between the materials’ porosities and mechanical strengths. The obtained results are interpreted by scanning electron microscopy analysis and hydration heat evolution to elucidate the major changes and their driving factors. Besides the benefits associated with the mitigation of autogenous shrinkage, the achieved results point to an adverse effect of supplementation with SAP on mechanical strength at an early age, and an even more pronounced increase at a later age. The employed scanning electron microscopy images, together with mercury-intrusion porosimetry data, depict distortion in the material porosity as a result of the filling of formed voids and the closing of open ends by swelled hydrogels. Only the minor benefit of a greater cross-linking density was obtained by the formation of dense structures and the gains in mechanical strength therefrom.

## 1. Introduction

Concrete, as the most used building material, suffers from various undesired effects such as shrinkage, expansion, and consequent cracking that may result in limited service life, undesired maintenance cost, and increase the risk of collapse [1]. Since the fundamental causes of crack formation have been described in the literature in sufficient detail, consequent action, aiming at mitigation of material deterioration associated with undesired shrinkage, still requires further research [2,3,4]. The conventional methods employed for shrinkage reduction are usually based on water-curing, steaming, and covering of the fresh mixtures. However, this approach cannot be used in all cases. Especially in the development of advanced types of concrete, particularly, high-performance concrete with a low water/cement ratio requires additional treatment to reduce the self-desiccation and consequent crack occurrence [5]. In this regard, internal curing has been found a progressive method for the mitigation of undesired shrinkage, the improvement of material durability, and for prolonging service life [6,7]. Today, internal curing is mostly secured by inorganic porous materials or chemical polymer admixtures, such as lightweight aggregate [8], rice husk ash [9], fly ash [10], cenospheres [11], or superabsorbent polymers (SAPs) [12]. As reported, the application of internal curing agents may increase the level of cement hydration and thus improve the hardening of the matrix. Among others, SAPs have attracted eminent attention as internal curing agents with a great swelling capability that allows the absorption of more than a hundred times its weight of water or aqueous solutions [13,14]. The principle of the internal curing maintained by SAPs is based on the continuous release of moisture during the curing period and also the compensation of internal moisture loss induced by the formation of cement hydration products. Water released from SAP particles has a beneficial effect on the degree of hydration, the magnitude of the reduction in shrinkage, and the degree to which crack occurrence is mitigated. To be specific, the utilization of SAPs in high-performance concrete (HPC) or even ultra-high-performance concrete (UHPC) has been found to be a very efficient method for shrinkage prevention [15,16,17]. Similarly, the above-mentioned materials can be utilized as self-healing agents that promote crack sealing. The principles of self-healing are based on swelling of calcium silicate hydration products triggered by additional water supply secured by lightweight aggregates, fibers, capsules, bacteria, or pozzolan admixtures [6,18,19,20,21].

However, SAPs’ application is accompanied by several drawbacks that hinder the potential utilization of high-performance building materials [22]. SAPs represent a heterogeneous group of materials that differ in several aspects; thus, materials’ parameters need to be investigated in detail to take into account potential undesired consequences. Among others, particle size, chemical composition, dosage method, pH sensitivity, swelling capability, desorption rate, etc. have a huge effect on sorption kinetics and strongly affects the fresh mixture workability, porosity formation, and hardened state properties [13,23,24,25]. To optimize the effect of SAPs, it is necessary to understand inner moisture migration in cement-based materials, as proposed by Danish et al. [18] or Yang et al. [26]. Specifically, the properties of the SAP-surrounding area need to be explored to provide a coherent overview applicable for advanced concrete design. In this regard, the adverse effects associated with impropriate SAP dosages need to be studied as well [27]. In the past, the kinetics of water, released from swelled SAPs into the cement matrix, has been studied with the help of nuclear magnetic resonance [28], concluding that significant differences can be found between selected SAPs regarding their time-dependent water release. With this relation in mind, SAPs’ moisture diffusion properties were investigated by Tan et al. [29], who described portlandite formation and the crystal size of alite induced by the addition of SAPs. The effects which accompany the formation of pore structures notwithstanding, absorption/desorption phenomenon and contributions therefrom to mechanical strength still require significant attention from researchers in order to overcome the present issues associated with SAPs’ application [30,31].

Besides the above-mentioned issues related to SAPs’ incorporation in cementitious materials, the utilization of SAPs also provides side benefits linked with improved freeze–thaw resistance, thanks to the formation of larger pores. This beneficial material performance was described in detail by Kim et al. [32], who achieved notable improvements in terms of material strengthening, which were secured by sufficient internal curing. The authors noted that this achievement could be further increased by the precisely tailored selection of SAPs and dosages thereof.

Based on the original purpose of SAP utilization—prolonging concrete structures’ service lives—the description of trends in material structure development and in mechanical performance represent a major topic, requiring comprehensive research. Despite the fact that the principle of self-healing is relatively well understood and widely discussed in the scientific literature [16,32,33], several questions still remain unanswered. Regardless of many research papers aimed at the determination of water’s distribution in cement matrices at various ages [34,35,36], the understanding of the effects of SAPs characteristics on the functional properties of cement still has significant gaps. It is of great significance to avoid the undesired phenomena associated with SAPs’ utilization and, concurrently, to elucidate the role of SAPs’ formation of microstructures in cement mortars.

Toward this end, in the present work the experimental analysis of selected SAPs’ effect on pore-size distribution, shrinkage mitigation, and the evolution of mechanical strength, as well as the distribution of the SAPs in the examined specimens, is presented. The obtained findings provide new insights into the design of advanced concrete types with improved self-healing capabilities, with emphasis on proper SAP dosing and selection.

## 2. Materials and Methods

### 2.1. Studied Materials

For this study, Portland cement CEM I 52.5 R (Heidelberg Cement, Praha, Czech Republic) was used. Details of the used cement in terms of its chemical composition and basic information are presented in Table 1. The specific surface area, as determined by a Blaine device, was about 512 m^2^/kg.

**Figure 1:**
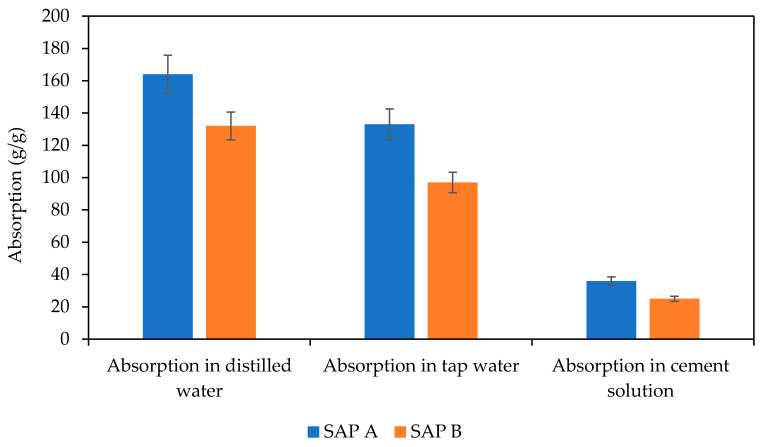
Absorption characteristics of studied SAPs.

Two grades of SAPs, differing in chemical composition and swelling, were used (Evonic, Essen, Germany). SAP A was based on sodium polyacrylate and had a mean particle size of 62 µm, with a higher degree of cross-linking density. SAP B consisted of acrylamide/acrylic acid and sodium salt and had an average particle diameter of 87 µm, with a lower degree of cross-linking density. The details of the SAPs’ swelling (see Figure 1) were determined with the help of the method presented in Fořt et al. [13]. Here, SAP samples with a weight of about approximately 1 g were placed into a teabag and immersed in the used solution (distilled water, tap water, cement solution) for 60 min. The results showed a representative mean value obtained from five independent experiments. For this experiment, the cement filtrate solution with a water/cement ratio of 0.5 was applied.

### 2.2. Mortars Design

Table 2 present the mix proportion used for the design of the cement mortars modified with selected SAPs in the following dosages: 0.3%; 0.6%, and 0.9% of the mean of the cement mass as established in preliminary tests. A/C describes the applied aggregate/cement ratio. Here, three grades of silica sand were used (0.3–0.8 mm; 0.6–0.8 mm; 1–4 mm) in a 1:1:1 ratio (Sklopísek Střeleč, Hrdoňovice, Czech Republic), which had a density of about 2645 kg/m^3^. The incorporation of SAPs adversely effects fresh mixtures’ rheology; thus, the reference mixture must be modified to preserve the mortar applicability. With consideration to recent advances in the field, it was necessary to carefully select the reference parameter to be used in comparisons of the designed mixtures. As revealed, keeping a constant w/c ratio is rather an inappropriate way to design SAP-modified mortars, since the applied SAPs will absorb a substantial part of the water and the real w/c will be lowered below the stated value [37]. Consequently, this effect may result in the undesired stiffness of the fresh mixture, the insufficient hydration of cement particles, and the distortion of its material properties [38]. To overcome issues accompanied by highly absorbing admixtures, a constant workability parameter was introduced as a constant reference parameter, instead of the w/c ratio, for mixture design. This approach has been established and verified in the works of Lee et al. [37] and Schrofl et al. [38], who used the same workability parameter to design SAP-modified binder mixtures.

To avoid the adverse effects of SAPs and maintain optimal workability levels, the parameter ‘constant flow-table diameter’ was applied in this study. In this regard, the w/c ratio used for the reference mixtures was adjusted in the SAP-modified mortars, to compensate for water consumed by the SAP particles. To keep the workability level as the constant parameter, the flow-table diameter of the reference mixture was measured (232 mm). Consequently, an extra amount of water (Ew) was added to the SAP-modified mortars to maintain a constant workability level. We assume that the majority of the water would be consumed by the SAPs; therefore, the observed changes in the material microstructure can be assigned to SAPs’ presence in the mixtures.

The samples were cast in molds, demolded after 1 day, and stored for 28 days, immersed in water. Samples were dried at 65 °C until reaching a steady-state mass and then subjected to the experimental procedures described in the following section. All performed experiments were done under laboratory conditions (21 °C/40%RH).

### 2.3. Experimental Methods

#### 2.3.1. Scanning Electron Microscopy

The microstructures of the studied cement mortar mixtures were studied by scanning electron microscopy (SEM) by using a field emission gun scanning electron microscope Merlin-ZEISS (FEGSEM, Zeiss, Jena, Germany) equipped with a secondary electron detector operating at an acceleration voltage of 15 kV, probe current of 300–800 pA, and a working distance of 6–18 mm. The chemical compositions of the materials’ surfaces were determined by energy-dispersive X-ray spectroscopy (EDS). SEM images were analyzed using the NIS-Elements software (Laboratory Imaging Ltd., Prague, Czech Republic). The vacuum-dried tested samples were mounted on aluminum stubs using double-sided conductive carbon tape and sputter-coated with gold/palladium using a Quorum SC7620 sputter coater (Quorum Technologies Ltd., Lewes, United Kingdom).

#### 2.3.2. X-ray Computed Tomography Analysis

In order to conduct the X-ray computed tomography (XCT) analysis of the cement mortars, samples of 10 × 10 × 10 mm were used. For this purpose, computed tomography equipment, with a multi-focus open tube and tungsten target (25–160 kV and 0.01–1.0 mA) YXLON COUGAR SMT (YXLON, Hudson, OH, USA), was used. The employed experimental equipment allowed 2D and 3D inspections with a geometric magnification of up to 10,000x. The VGStudio MAX and AVIZO software (Volume Graphics, Heidelberg, Germany) was used for the realization and analysis of the 3D reconstructions.

#### 2.3.3. Hydration Heat Evolution

The hydration heat evolution, as well as the total heat evolution during the hydration period of the cements, together with SAP, was studied by an isothermal calorimeter at room temperature (25 °C). The conduction calorimeter TAM AIR (TA Instruments, New Castle, DE, USA) was employed for the determination of hydration heat development. The measurement was carried out with a 5 g sample of the dry mixture in a copper vessel. After embedding the copper vessel with the studied mixture into the stabilized calorimeter, a weighted syringe with water was placed near the reaction vessel with a plastic tubule, intended for paste-mixing. A calorimeter with a solid sample and liquid components was stabilized at the operating temperature (25 °C). After about 1 h of stabilization, the calorimeter was opened, and water was injected into the vessel with the studied mixture. The paste was mixed for 30 s by rotation of the plastic tubule mixer, afterwards, the vessel was sealed with a rubber plug. The duration of the experiment was 80 h.

#### 2.3.4. Shrinkage

The shrinkage test was carried out by using 40 mm × 40 mm × 160 mm prismatic specimens for 84 days after the final setting according to ČSN EN 12390-16. Specimens were placed in the climatic chamber (21 °C, 50%RH) and periodically measured by using a non-contact measuring device.

#### 2.3.5. Basic Physical Characteristics

The basic material properties, including material porosity, bulk, and matrix density, were determined. The matrix density was obtained by helium pycnometry by using an ATC EVO device (ThermoScientific, Waltham, MA, USA). The bulk density was calculated on the basis of the gravimetric measurement. With the help of the bulk and matrix densities of studied specimens, total open porosities were determined. Measurements were conducted on five cubic samples with a side length of 50 mm. The accuracy of the gas volume measurement using this device is ±0.01% from the measured value, whereas the accuracy of the used analytical balances is ±0.0001 g. The bulk-density uncertainty was 5.3% and 3% for the matrices’ densities.

#### 2.3.6. Mercury Intrusion Porosimetry

The pore-size distribution was characterized by using the mercury-intrusion porosimetry devices, Pascal 140 and Pascal 440 (ThermoScientific, Waltham, MA, USA). Tested samples were first dried, to reach a constant mass, and, thereafter, placed into a glass container, which was filled with mercury. Within the experiment, the pressure was gradually increased from 100 kPa up to 400 kPa to force the mercury’s penetration of the pores of the studied samples.

#### 2.3.7. Mechanical Properties

Mechanical strength, in the sense of the compressive and flexural strength after 7, 28, and 180 days, was estimated according to ČSN EN 206-8. Immediately afterward, the prismatic samples were demolded and placed into water to secure sufficient water curing. After 7 days, the first set of samples were used for the determination of flexural and compressive strength. A similar procedure was repeated after 28 days. After this period, the rest of the samples were placed under laboratory conditions (21 °C, 50%RH) until the end of the 180-day period. Flexural strength was measured by a three-point bending test on three specimens of dimensions of 40 mm × 40 mm × 160 mm. Subsequently, compressive strength was measured for the left-over specimens from the flexural strength test.

## 3. Results and Discussion

**Figure 2:**
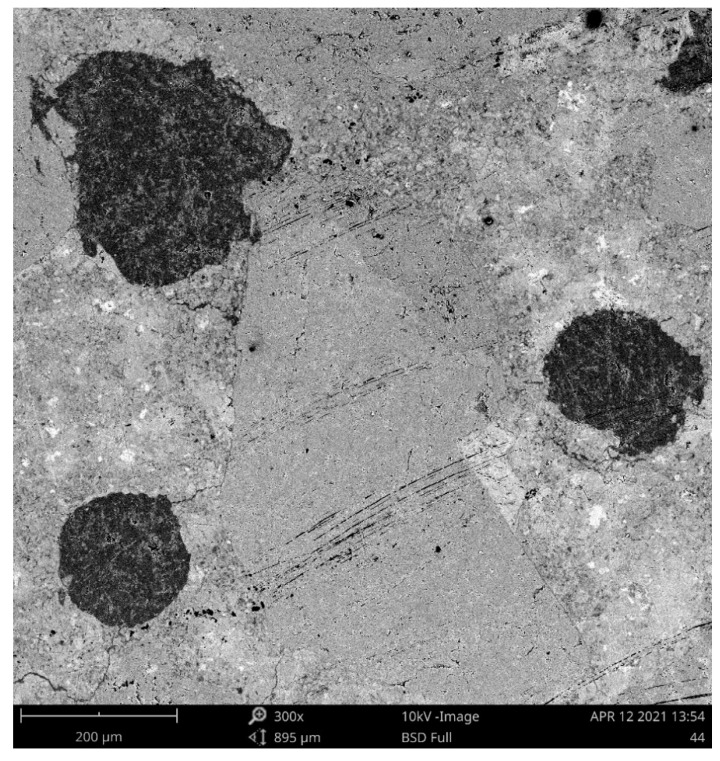
SEM image of SAP particles embedded in a mortar structure (A3 mixture).

**Figure 3:**
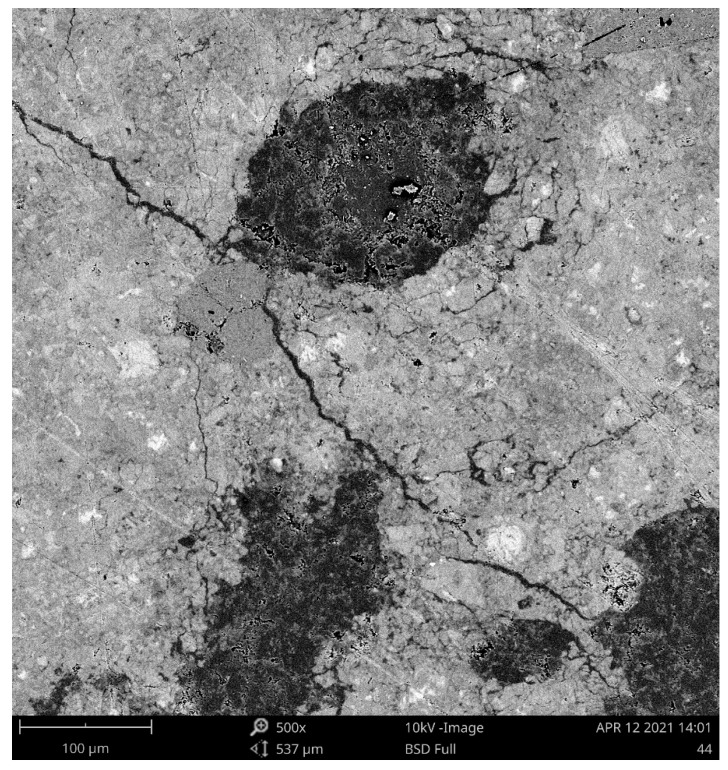
SEM image of the cracks that formed around SAP particles (A3 mixture).

**Figure 4:**
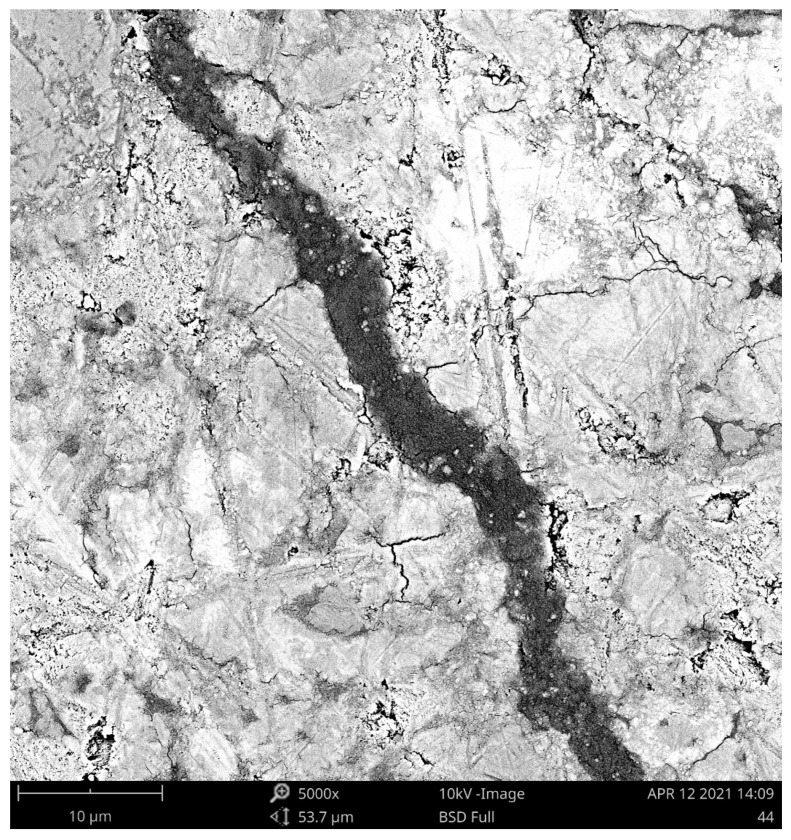
SEM image of a sealed crack (A3 mixture).

SEM analysis was employed to depict the details of the SAP particles embedded in the cement mortar structures and, concurrently, to provide a better description of the SAP particles’ surroundings. Contrary to the commonly used concrete admixtures, swollen SAP particles are flexible in shape, and thus can fill very irregular voids very tightly. Taking into account SAPs’ remarkable swelling capabilities, the formed voids can also be several times larger than the average SAP diameter in a dry state, according to the number of dissolved cations in the water. As one can see, the diameters of the SAP particles were approximately doubled or tripled and accompanied by the formation of microcracks near them (see Figure 2). In greater detail, Figure 3 shows the lengths and the diameters of the identified cracks. Consequently, Figure 4 depicts the details of sealed cracks connected to SAP particles. As visible, the SAP particles expanded into the surrounding cracks and closed them entirely. First, this finding may help in understanding the results of the porosity analysis, and, second, it may contribute to describing affected zones, which is currently the subject of detailed research.

**Figure 5:**
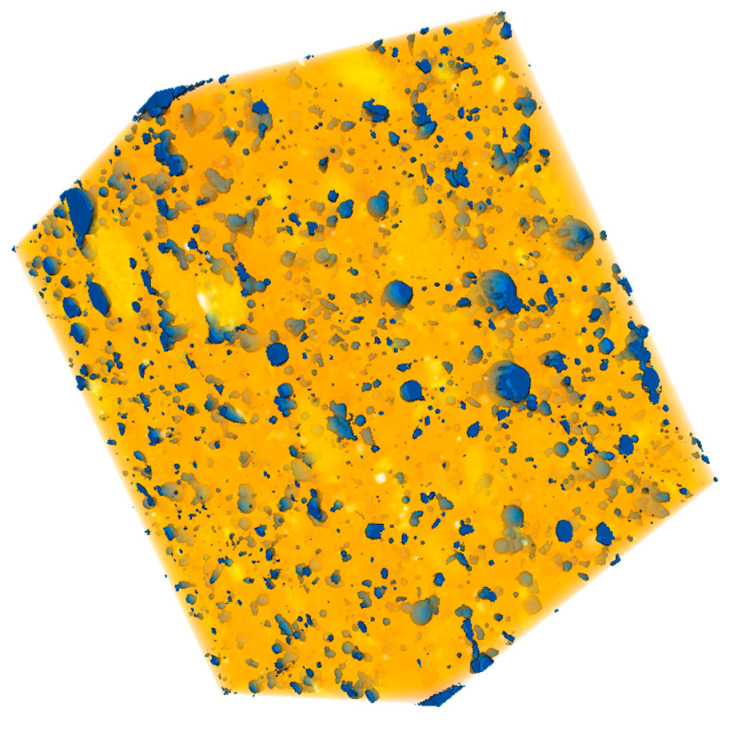
3D XCT render of the A3 mixture sample (10 × 10 × 10 mm) with revealed SAP particles (blue color).

The utilization of SAPs in cement mortar samples is often accompanied by undesired SAP-particle clumping that diminishes the self-healing performance and creates non-compact areas. Afterward, adverse effects on functional material properties, such as the loss of mechanical strength, can be expected. To verify the uniform distribution of SAP particles, XCT measurement was employed with the 3D reconstruction of the cement mortar sample (A3 mixture), and the SAP particles’ distributions were obtained as shown in Figure 5. As can be appreciated, these particles were homogeneously distributed and no significant clumping was revealed. While the particle size distribution curve of the used SAPs [35] refers to a relatively close interval of particle size, significant diameter variations were observed. Therefore, volume changes of the used SAPs, due to their swelling, subsequently caused more significant changes than expected on the basis of the mixture design. Besides this fact, the characterization of SAPs, using the data provided by the manufacturer, did not reflect the real rate of swelling, which differed significantly, depending on the solution in which the SAP was used. In this sense, SAPs differ from other admixtures, which usually do not show such significant volume changes. Thus, the characterization of SAPs based on their initial particle size is not significantly relevant.

**Figure 6:**
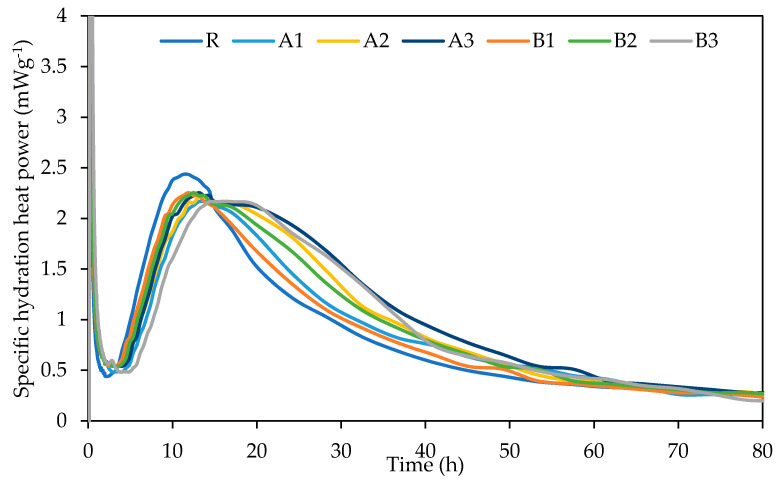
Hydration heat development.

The cumulative hydration heat evolution curves are plotted in Figure 6. Here, the different amounts of hydration heat over time can be distinguished. Provided results reveal that the main peak of the hydration curve was lowered in line with increasing SAP dosage. This effect is more evident for SAP A mixtures. On the contrary, the hydration period was extended proportionally, in contrast with the reference mixture. Such finding could be aligned to an additional water supply to the cement during a later stage of the hydration reaction, thus SAP incorporation prolongs the hydration reaction and secures more thorough cement hydration [39]. This effect is visible also in Figure 6, as the increased cumulative heat evolution over time, as compared with the reference mixture at a later age, despite very similar evolution within the first 12 h. Thus, the most noticeable changes were obtained by the A3 mixture (followed by B3), particularly by those mortars with the highest SAP content. The observed differences between the selected SAPs refer to different water sorption characteristics, in other words, also to the discrepancy during desorption, accompanied by improved and prolonged curing. The significant impact of the SAP addition on the degree of cement hydration has also been remarked upon by Justs et al. [40].

**Figure 7:**
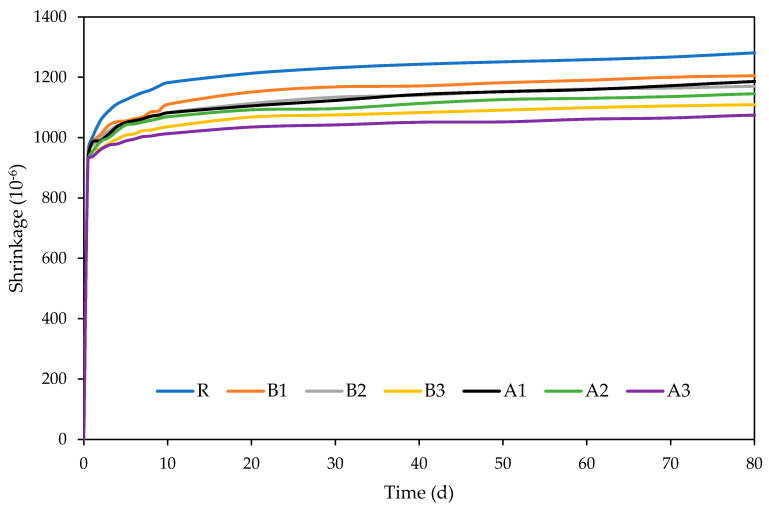
Shrinkage development of studied cement mortars.

The contribution of the applied SAPs to the mitigation of cement mortar shrinkage is shown in Figure 7. As shown, the total shrinkage of the reference cement mortar was most distinct in the first days, with a decreasing tendency at later ages. The addition of SAP resulted in the mitigation of the autogenous shrinkage as compared to reference mortar. The most beneficial results were obtained for the A3 mixture with the highest SAP dosage, while the lower dosages lowered the shrinkage proportionally. However, SAP B did not result in such a distinct reduction in material shrinkage during the curing period. The most probable explanation can be found in its lower water absorption capacity and thus consequent desorption capability, which provides sufficient internal curing and internal water supply. These findings agree with the findings of Urgessa et al. [41], who correlated the results of autogenous shrinkage with internal relative humidity for low w/c mortars. Here, the crosslinking density was also found as a beneficial parameter that may contribute to the reduction in the autogenous shrinkage due to the higher the elastic retraction squeezing rapidly out the water absorbed in SAP to the matrix. The performed investigations in this field refer to an almost linear relationship between the development internal humidity and autogenous shrinkage. In this regard, SAPs’ application can be viewed as a creating a favorable admixture, reducing the undesired volumetric changes that accompany self-desiccation.

The basic material properties, such as total open porosity, bulk, and matrix density, after 28 days, are listed in Table 3. As one can see, only minor variations can be noted in the matrix densities for all the designed cement mortars. On the contrary, more pronounced changes, driven by gradually increased SAP content, were achieved. Specifically, the most significant drop in bulk density, of about 10%, was obtained from the use of 0.9% SAP A, while SAP B’s application resulted in a bulk density reduction of 7.5%. Similarly, lower SAP dosages induced a proportional decrease in bulk density. Thereafter, the calculated total open porosity was affected in the same way, i.e., the lowest porosity can be attributed to reference mixture, followed by mortars with lower SAP dosages. As visible, the use of SAP B affected the basic material properties of cement mortars to a lesser extent as compared with SAP A. This phenomenon refers to the decreased swelling of SAP B and thus reduced extra water dosage [42]. Similar effects of the SAP content on void formation were described by several researchers, who used similar portions of SAP for the cement mortar or concrete modifications.

In order to access the long-term effects of used SAPs on the material performance of cement mortars, their basic material properties were also determined after 180 days (Table 4). Major changes in the material properties arose from the increase in the bulk density and consequent reduction in the total open porosity. This beneficial performance is driven by extended internal curing, secured by the continuous hydration of unhydrated cement particles, and the sealing of cracks. Moreover, SAPs are widely considered autogenous-mitigation agents, hindering internal stress. As visible in the above-presented Figure 7, the sealing of cracks reduces free voids and may lead to the full-healing of such formed cracks. As shown, this effect also led to a reduction in total open porosity, by as much as 15.5% for the reference mortar. Notwithstanding, a more distinct drop in pore volume was observed for mortars with higher SAP content, which agrees with the results of Tan et al. [43] and Song et al. [44]. On the other hand, the effect of materials’ maturation did not induce any substantial changes in matrix density.

**Figure 8:**
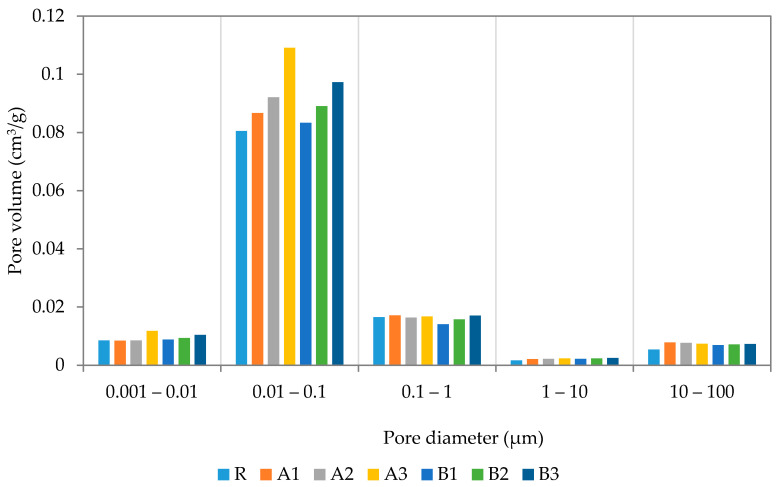
MIP of designed cement mortars at 28 days.

More detailed information about pore size distribution was obtained by mercury intrusion porosimetry (MIP). The pore size distribution curves after 28 days are presented in Figure 8. As recorded, the application of both SAP types resulted in increased pore volumes as compared with the reference mixture, proportionally to their applied dosages. The inner structures of the cement mortars were affected in a similar way by both used SAPs, while only very limited differences could be noted. In general, more pronounced mercury retention was observed for samples with SAP A, thus the greater water swelling therefrom can be viewed as a crucial aspect [45]. Major changes were observed in the pore range of 10—100 nm, while the rest did not reach distinct modifications. The MIP results correspond well with the results of the basic material properties, as a result of changes in the material workability and the extra water dosage. A similar material response was studied by Yang et al. [46], who focused on pore distributions and the formation of affected zones around SAP particles, in particular. The slightly higher volume of larger pores revealed in the modified mixtures is related to the desorption of SAP cores.

**Figure 9:**
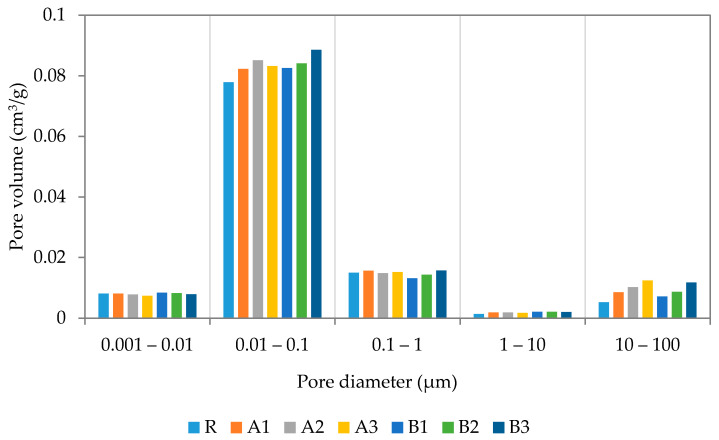
MIP of the designed cement mortars at 180 days.

Pore distribution curves were determined after 180 days of sample curing and refer to significant changes in inner structure (see Figure 9). The continuous internal curing secured by SAPs substantially reduced pore volumes between 10 nm and 100 nm and, contrarily, slightly increased the volume of larger pores. In summary, the total volume of open pores was reduced in line with expectations based on the research of Kanellopoulou et al. [47] and Klemm et al. [48]. It should be noted that the performed analysis was based on Washburn’s model, which assumes the cylindrical shape of pores with an open end and circular cross-sections. However, this condition does not reflect the real pore structure and may distort the image of the pore structure. This note is important due to the possible formation of closed pores consisting of entrapped SAP particles. Considering the self-healing capability of SAP particles, the pore volume reduction between 28 and 180 days could be partially assigned to the closing of ends of interconnected pores by continuous hydration [49]. This investigation, aimed at the formation of pore structures, concludes that the utilization of SAPs is responsible for the reduction in material permeability. This fact correlates with the above-presented SEM images showing the sealed cracks being connected to the surface. On the other hand, water released from SAPs during desorption is probably responsible for the shift in the larger pores’ formation. Significant differences, recently published, can be attributed to the influence of particle size, materials used, treatment conditions applied water/cement ratio, etc [44,47,48]. It should also be noted that the use of MIP analysis cannot be seen as the most appropriate analysis to describe the internal structure of SAP-modified materials; however, it is sufficient to describe trends therein.

**Figure 10:**
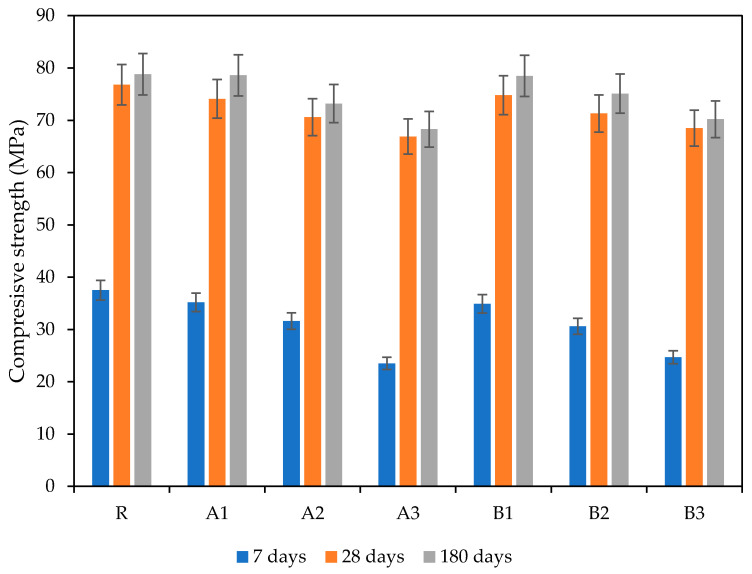
Time development of compressive strength.

**Figure 11:**
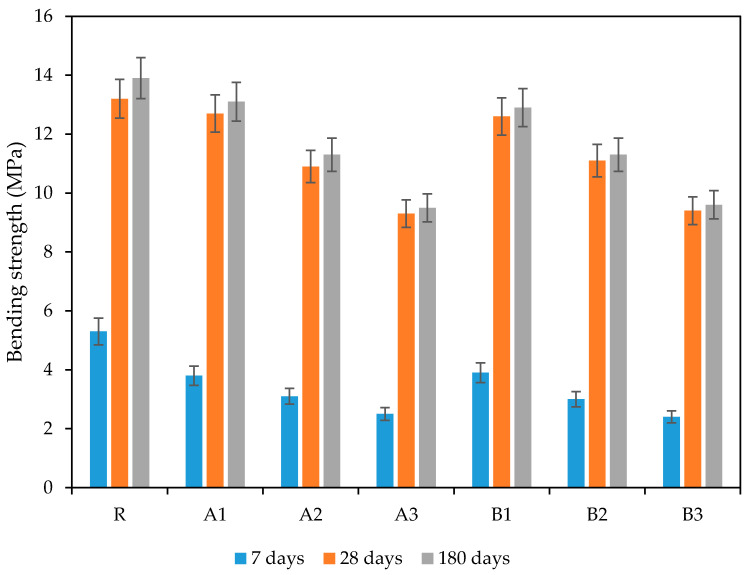
Time development of bending strength.

Figure 10 and Figure 11 show the changes in the compressive and bending strength induced by used SAP admixtures at different ages (7 days, 28 days, 180 days). As can be seen, the incorporation of SAPs resulted in negative effects on mechanical performance. This phenomenon was more distinct at the yearly age, since SAP particles’ swelling damaged the structure of the mortar by forming voids. Additionally, hydrogel-based SAP particles further decreased the compressive strength of the mortars, which differs from other frequently used additives. This effect was further shifted, in line with increasing SAP dosages proportionally in both SAP types, though the effect of cross-linking density cannot be clearly evaluated.

The further maturing of the samples slightly diminished the initial differences between particular mixtures, therefore, the benefits associated with improved internal curing driven by SAP deswelling can be observed. The reference mixture achieved the best mechanical performance (about almost 80 MPa) despite a more pronounced improvement in the SAP-modified mixtures. The results obtained after 180 days showed similar changes to those described at 28 days, such the SAP played an important role as an internal curing agent that promoted the further hydration of unhydrated particles [45,46]. However, the obtained decrease in mechanical strength would be even more distinct were higher w/c applied. As the results from Farzanian et al. [50] show, the application of a higher w/c ratio may worsen mechanical performance with a higher probability, due to the formation of larger voids. Moreover, the effect of internal curing is meaningful for mixtures with w/c about 0.4, or even lower, since 0.42 is the accepted threshold value sufficient for cement particles’ hydration. The utilization of SAPs represents a very complex issue, with many variables needing to be taken into account when presenting such data. Kanellopoulou et al. [47] proved that the precise selection of a suitable SAP type during the design stage, which considers structure, particle size, and shape, could mitigate the adverse effects commonly associated with SAPs’ use [51,52].

The slightly higher reduction in the compressive strength that occurred in cement mortar samples containing SAP can be attributed to higher porosity—to the number of macropores, in particular. The obtained results for the reference mixture correlate with trends in total open porosity development and are in good agreement with the conclusions of Abed and Nemes [53]. The results of the flexural strength study showed the same trends, in terms of the effect of SAP particles, from SAP type and dose. Besides an SAP’s characteristics, the curing conditions used, and the method of dosing may also contribute to the preservation of mechanical performance, since these substantial effect porosity formation.

**Figure 12:**
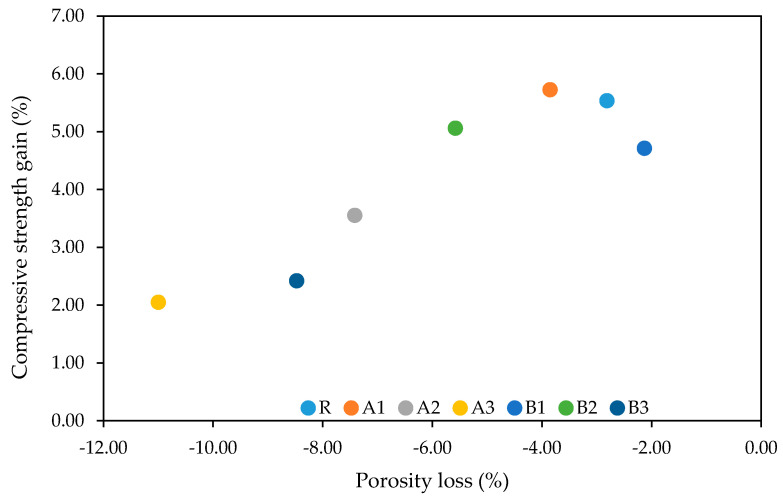
Relationship between porosity loss and compressive strength gain.

However, it must also be noted that the relationship between porosity and mechanical resistance is dependent on the method used. As depicted in Figure 12, the dependency between the change in compressive strength and the difference between the total open porosity, at 28 and 180 days, does not correspond to commonly accepted principles [54]. While the most expressive change belongs to mortars with the highest SAP dosages, only minor improvements in compressive strength were achieved. The cause of this apparent disproportion is not clear and requires further investigation.

A possible explanation may lie in the healing of cracks and small pores. As follows from the SEM images, the sealing capability of the SAP particles resulted in the closing of connecting channels. Taking into account the promotion of the subsequent hydration of cement particles in the SAP’s surroundings, matrix densification makes it difficult to describe the volume of pores by MIP analysis. In this regard, the effectiveness of the MIP analysis was significantly reduced. Moreover, the formed voids filled by swollen SAP particles worsened the mechanical performance substantially.

## 4. Conclusions

A series of experiments were carried out to assess the effects of different dosages and types of superabsorbent polymers on the performance of cement mortars at 28 and 180 days. For this purpose, two grades of superabsorbent polymers, with different crosslinking densities and particle sizes, were selected and further used for the modification of cement mortar by 0.3%; 0.6%; and 0.9%. The revealed findings follow:

- The application of SAPs lowered the main hydration peak at 10 h, while the cumulative heat evolution of the SAP-modified pastes dominated over the reference mixture, in the reverse order.
- The effect of cross-linking density was rather minor, in terms of mechanical and bending strengths. The slight variation in the observed material properties can be attributed to the reduced deswelling rate by superabsorbents with lower cross-linking densities.
- SEM analysis, performed together with XCT, revealed a regular distribution of incorporated SAP particles with only minor clumping. This finding suggests that the benefits associated with SAPs’ application, such as mitigation of autogenous shrinkage and crack self-healing, occur proportionally in the material matrix. Furthermore, the cracks formed in the SAP particles’ surroundings were filled by the swollen hydrogel and promoted self-healing, due to improved internal curing.
- The benefits associated with the mitigation of autogenous shrinkage varied positively with dosage. Slightly more favorable results were obtained for the mixture modified by SAPs with higher cross-linking densities.
- The application of superabsorbent polymers increased pore volume in the range of 10–100 nm at 28 days, however, at 180 days, the observed change in porosity was reduced to the almost same level as the reference mixture. This phenomenon can be attributed to the formation of affected zones around the SAP particles, which was responsible for the closing of open pores. In this regard, MIP analysis may result in a significant distortion of real porosity values, due to decreased penetration of older samples. Moreover, swollen SAPs in the inner structure of mortars fill the voids formed by the hydrogel, however, such filled voids further deteriorate the mechanical strength.

In the light of the obtained findings, further research, aimed at detailed time-dependent volumetric changes of SAP particles during the maturing of cement mortars, is acutely required.

## Figures and Tables

**Table 1:** Chemical composition of the used cement.

SiO_2_	CaO	Al_2_O_3_	Fe_2_O_3_	Na_2_O	K_2_O	SO_3_	Cl^−^	MgO	LOI
21.78	65.49	4.13	0.41	0.32	0.33	3.32	0.01	1.35	2.36

**Table 2:** Composition of designed cement mortars.

	Cement (kg/m^3^)	w/c	SP (%)	A/C	SAP (%)	Ew (%)
R	500	0.3	0.3	2	0	0
A1					0.3	2.3
A2					0.6	5.87
A3					0.9	11.6
B1					0.3	1.8
B2					0.6	5.37
B3					0.9	10.3

**Table 3:** Basic material properties of designed cement mortars at 28 days.

Mixture	Matrix Density (kg/m^3^)	Bulk Density (kg/m^3^)	Open Porosity (%)
R	2715 ± 13	2280 ± 21	16.0 ± 1.2
A1	2714 ± 15	2229 ± 18	17.9 ± 1.4
A2	2712 ± 15	2165 ± 19	20.2 ± 1.1
A3	2709 ± 14	2087 ± 16	22.9 ± 1. 3
B1	2715 ± 10	2238 ± 17	17.6 ± 1.4
B2	2714 ± 13	2181 ± 18	19.6 ± 1.1
B3	2710 ± 16	2113 ± 15	22.1 ± 1.2

**Table 4:** Basic material properties of the designed cement mortars at 180 days.

Mixture	Matrix Density (kg/m^3^)	Bulk Density (kg/m^3^)	Open Porosity (%)
R	2723 ± 11	2299 ± 18	15.6 ± 1.0
A1	2718 ± 14	2251 ± 19	17.2 ± 1.2
A2	2715 ± 12	2208 ± 21	18.7 ± 1.1
A3	2711 ± 12	2157 ± 17	20.4 ± 1.3
B1	2716 ± 13	2249 ± 18	17.2 ± 1.1
B2	2718 ± 14	2214 ± 13	18.5 ± 1.0
B3	2713 ± 15	2166 ± 17	20.2 ± 1.2

## Data Availability

The data presented in this study are available on request from the corresponding author.

## References

[1] de Brito J., Kurda R. (2021). The past and future of sustainable concrete: A critical review and new strategies on cement-based materials. J. Clean. Prod..

[2] Cabeza L.F., Boquera L., Chàfer M., Vérez D. (2021). Embodied energy and embodied carbon of structural building materials: Worldwide progress and barriers through literature map analysis. Energy Build..

[3] Jittin V., Rithuparna R., Bahurudeen A., Pachiappan B. (2021). Synergistic use of typical agricultural and industrial by-products for ternary cement: A pathway for locally available resource utilisation. J. Clean. Prod..

[4] Gupta N., Siddique R., Belarbi R. (2021). Sustainable and Greener Self-Compacting Concrete incorporating Industrial By-Products: A Review. J. Clean. Prod..

[5] Wu L., Farzadnia N., Shi C., Zhang Z., Wang H. (2017). Autogenous shrinkage of high performance concrete: A review. Constr. Build. Mater..

[6] Yang L., Shi C., Liu J., Wu Z. (2021). Factors affecting the effectiveness of internal curing: A review. Constr. Build. Mater..

[7] Liu J., Farzadnia N., Khayat K.H., Shi C. (2021). Effects of SAP characteristics on internal curing of UHPC matrix. Constr. Build. Mater..

[8] Shah S.N., Mo K.H., Yap S.P., Yang J., Ling T.-C. (2021). Lightweight foamed concrete as a promising avenue for incorporating waste materials: A review. Resour. Conserv. Recycl..

[9] Hossain S.S., Mathur L., Majhi M.R., Roy P.K. (2018). Manufacturing of green building brick: Recycling of waste for construction purpose. J. Mater. Cycles Waste Manag..

[10] Fořt J., Šál J., Žák J., Černý R. (2020). Assessment of Wood-Based Fly Ash as Alternative Cement Replacement. Sustainability.

[11] Baloch H., Usman M., Rizwan S.A., Hanif A. (2019). Properties enhancement of super absorbent polymer (SAP) incorporated self-compacting cement pastes modified by nano silica (NS) addition. Constr. Build. Mater..

[12] Zohuriaan-Mehr M.J., Kabiri K. (2008). Superabsorbent polymer materials: A review. Iran. Polym. J..

[13] Fořt J., Migas P., Černý R. (2020). Effect of Absorptivity of Superabsorbent Polymers on Design of Cement Mortars. Materials.

[14] Yang J., Sun Z., Zhao Y., Ji Y., Li B. (2020). The Water Absorption-release of Superabsorbent Polymers in Fresh Cement Paste: An NMR Study. J. Adv. Concr. Technol..

[15] Liu J.H., Farzadnia N., Shi C.J., Ma X.W. (2019). Effects of superabsorbent polymer on shrinkage properties of ultra-high strength concrete under drying condition. Constr. Build. Mater..

[16] Olawuyi B.J., Babafemi A.J., Boshoff W.P. (2021). Early-age and long-term strength development of high-performance concrete with SAP. Constr. Build. Mater..

[17] Fan J., Shen A., Guo Y., Zhao M., Yang X., Wang X. (2020). Evaluation of the shrinkage and fracture properties of hybrid Fiber-Reinforced SAP modified concrete. Constr. Build. Mater..

[18] Danish A., Mosaberpanah M.A., Salim M.U. (2020). Robust evaluation of superabsorbent polymers as an internal curing agent in cementitious composites. J. Mater. Sci..

[19] Aparicio S., Hernández M., Anaya J. (2020). Influence of environmental conditions on concrete manufactured with recycled and steel slag aggregates at early ages and long term. Constr. Build. Mater..

[20] Hawreen A., Bogas J. (2019). Creep, shrinkage and mechanical properties of concrete reinforced with different types of carbon nanotubes. Constr. Build. Mater..

[21] Ferraro A., Colangelo F., Farina I., Race M., Cioffi R., Cheeseman C., Fabbricino M. (2021). Cold-bonding process for treatment and reuse of waste materials: Technical designs and applications of pelletized products. Crit. Rev. Environ. Sci. Technol..

[22] Fernandez C.A., Correa M., Nguyen M.-T., Rod K.A., Dai G.L., Cosimbescu L., Rousseau R., Glezakou V.-A. (2020). Progress and challenges in self-healing cementitious materials. J. Mater. Sci..

[23] Guo Y., Zhang P., Ding H., Le C. (2020). Experimental Study on the Permeability of SAP Modified Concrete. Materials.

[24] Chindasiriphan P., Yokota H., Pimpakan P. (2020). Effect of fly ash and superabsorbent polymer on concrete self-healing ability. Constr. Build. Mater..

[25] Li D., Chen B., Chen X., Fu B., Wei H., Xiang X. (2020). Synergetic effect of superabsorbent polymer (SAP) and crystalline admixture (CA) on mortar macro-crack healing. Constr. Build. Mater..

[26] Yang H., Liu J., Jia X., Zhou Y., Ji H. (2020). Influence of NaCl concentrations on the crack-sealing behavior of superabsorbent polymers in cementitious materials. Constr. Build. Mater..

[27] Wang F., Yang J., Hu S., Li X., Cheng H. (2016). Influence of superabsorbent polymers on the surrounding cement paste. Cem. Concr. Res..

[28] Snoeck D., Pel L., De Belie N. (2019). Comparison of different techniques to study the nanostructure and the microstructure of cementitious materials with and without superabsorbent polymers. Constr. Build. Mater..

[29] Tan Y., Lu X., He R., Chen H., Wang Z. (2021). Influence of superabsorbent polymers (SAPs) type and particle size on the performance of surrounding cement-based materials. Constr. Build. Mater..

[30] He Z., Shen A., Guo Y., Lyu Z., Li D., Qin X., Zhao M., Wang Z. (2019). Cement-based materials modified with superabsorbent polymers: A review. Constr. Build. Mater..

[31] Schröfl C., Snoeck D., Mechtcherine V. (2017). A review of characterisation methods for superabsorbent polymer (SAP) samples to be used in cement-based construction materials: Report of the RILEM TC 260-RSC. Mater. Struct..

[32] Kim I.S., Choi S.Y., Choi Y.S., Yang E.I. (2021). An experimental study on absorptivity measurement of superabsorbent polymers (SAP) and effect of SAP on freeze-thaw resistance in mortar specimen. Constr. Build. Mater..

[33] Agostinho L.B., Alexandre D.P., da Silva E.F., Toledo R.D. (2021). Rheological study of Portland cement pastes modified with superabsorbent polymer and nanosilica. J. Build. Eng..

[34] Zhang B., Li Q., Ma R., Niu X., Yang L., Hu Y., Zhang J. (2021). The Influence of a Novel Hydrophobic Agent on the Internal Defect and Multi-Scale Pore Structure of Concrete. Materials.

[35] Kalinowski M., Woyciechowski P.P., Sokołowska J. (2020). Effect of mechanically-induced fragmentation of polyacrylic superabsorbent polymer (SAP) hydrogel on the properties of cement composites. Constr. Build. Mater..

[36] Lefever G., Aggelis D.G., De Belie N., Raes M., Hauffman T., Van Hemelrijck D., Snoeck D. (2020). The Influence of Superabsorbent Polymers and Nanosilica on the Hydration Process and Microstructure of Cementitious Mixtures. Materials.

[37] Lee H., Wong H., Buenfeld N. (2016). Self-sealing of cracks in concrete using superabsorbent polymers. Cem. Concr. Res..

[38] Schröfl C., Mechtcherine V., Gorges M. (2012). Relation between the molecular structure and the efficiency of superabsorbent polymers (SAP) as concrete admixture to mitigate autogenous shrinkage. Cem. Concr. Res..

[39] Fořt J., Doleželová M., Kočí V., Černý R. (2021). Functional Properties of SAP-Based Humidity Control Plasters. Polymers.

[40] Justs J., Wyrzykowski M., Winnefeld F., Bajare D., Lura P. (2014). Influence of superabsorbent polymers on hydration of cement pastes with low water-to-binder ratio. J. Therm. Anal. Calorim..

[41] Urgessa G., Choi K.-B., Yeon J.H. (2018). Internal Relative Humidity, Autogenous Shrinkage, and Strength of Cement Mortar Modified with Superabsorbent Polymers. Polymers.

[42] Ma X., Yuan Q., Liu J., Shi C. (2019). Effect of water absorption of SAP on the rheological properties of cement-based materials with ultra-low w/b ratio. Constr. Build. Mater..

[43] Tan Y., Chen H., Wang Z., Xue C., He R. (2019). Performances of Cement Mortar Incorporating Superabsorbent Polymer (SAP) Using Different Dosing Methods. Materials.

[44] Song C., Choi Y.C., Choi S. (2016). Effect of internal curing by superabsorbent polymers—Internal relative humidity and autogenous shrinkage of alkali-activated slag mortars. Constr. Build. Mater..

[45] Farzanian K., Ghahremaninezhad A. (2018). Desorption of superabsorbent hydrogels with varied chemical compositions in cementitious materials. Mater. Struct..

[46] Yang J., Wang F. (2019). Influence of assumed absorption capacity of superabsorbent polymers on the microstructure and performance of cement mortars. Constr. Build. Mater..

[47] Kanellopoulou I.A., Kartsonakis I.A., Charitidis C.A. (2021). The Effect of Superabsorbent Polymers on the Microstructure and Self-Healing Properties of Cementitious-Based Composite Materials. Appl. Sci..

[48] Klemm A.J., Sikora K.S. (2013). The effect of Superabsorbent Polymers (SAP) on microstructure and mechanical properties of fly ash cementitious mortars. Constr. Build. Mater..

[49] Sun B., Wu H., Song W., Li Z., Yu J. (2019). Design methodology and mechanical properties of Superabsorbent Polymer (SAP) cement-based materials. Constr. Build. Mater..

[50] Farzanian K., Teixeira K.P., Rocha I.P., Carneiro L.D.S., Ghahremaninezhad A. (2016). The mechanical strength, degree of hydration, and electrical resistivity of cement pastes modified with superabsorbent polymers. Constr. Build. Mater..

[51] Sun B., Wu H., Song W., Li Z., Yu J. (2020). Hydration, microstructure and autogenous shrinkage behaviors of cement mortars by addition of superabsorbent polymers. Front. Struct. Civ. Eng..

[52] Dang J., Zhao J., Du Z. (2017). Effect of Superabsorbent Polymer on the Properties of Concrete. Polymers.

[53] Abed M., Nemes R. (2019). Long-term durability of self-compacting high-performance concrete produced with waste materials. Constr. Build. Mater..

[54] Popovics S. (1990). Analysis of the concrete strength versus water-cement ratio relationship. Mater. J..

